# An International Aging Research Collaboration During The COVID-19 Crisis: Mitigating Global Health Consequences

**DOI:** 10.1093/geroni/igab046.544

**Published:** 2021-12-17

**Authors:** Wayne Chong, Rick Kwan, Inthira Roopsawang, Ramraj Gautam, W Q Lou Vivian, Ladda Thiamwong

**Affiliations:** 1 Nanyang Technological University, Singapore, Not Applicable, Singapore; 2 The Hong Kong Polytechnic University, Hong Kong, Hong Kong; 3 Mahidol University, Bangkok, Thailand; 4 UMass Lowell, Lowell, Massachusetts, United States; 5 The University of Hong Kong, Hong Kong, Not Applicable, Hong Kong; 6 College of Nursing, University of Central Florida, Orlando, Florida, United States

## Abstract

There are several reasons for forming an aging international research collaboration; however, creating a successful and productive research team during the global crisis may require extensive planning and efforts. Our team consists of ten scholars from five countries, including Hong Kong, Nepal, Singapore, Thailand, and the United States. To accomplish this initiative, we employ ten simple rules for establishing international research collaborations proposed by R. de Grijs (2015). We aim to examine impacts of the pandemic on physical activity, frailty, falls, depression and social networks in diverse older adults. We collect data by online survey and/or face-to –face survey using questionnaires including fear of the COVID, face mask use, Social Network, Rapid Assessment of Physical Activity, a simple frailty questionnaire, CDC fall risk checklist, short Fall-Efficacy Scale International and Patient Health Questionnaire-9. Topics of discussion included: research progression, lessons learned and barriers to international collaboration during the COVID-19 crisis.

